# From Growth Mindset to Grit in Chinese Schools: The Mediating Roles of Learning Motivations

**DOI:** 10.3389/fpsyg.2018.02007

**Published:** 2018-10-18

**Authors:** Yukun Zhao, Gengfeng Niu, Hanchao Hou, Guang Zeng, Liying Xu, Kaiping Peng, Feng Yu

**Affiliations:** ^1^Department of Psychology, Tsinghua University, Beijing, China; ^2^Institute of Social Psychology, Xi’an Jiaotong University, Xi’an, China; ^3^Centre for Positive Psychology, University of Melbourne, Parkville, VIC, Australia

**Keywords:** growth mindset, learning motivations, grit, positive education, self-determination theory

## Abstract

Growth mindset and grit have attracted much attention in educational research recently. Yet the underlying mechanisms that relate these variables to each other as well as to other variables remain largely unclear. This study investigates the relationships among growth mindset, learning motivations, and grit. We recruited a total of 1,842 students (884 males and 958 females) from third to ninth grade in a Chinese city. Results from the structural equation model analyzing the students’ responses showed that learning motivations partially mediate the relationship between growth mindset and grit. Specifically, intrinsic motivation and identified regulation of extrinsic motivation are positively associated with growth mindset and grit, while external regulation of extrinsic motivation is negatively associated with them. Additionally, introjected regulation of extrinsic motivation is uncorrelated with these two variables. This study furthers the understanding of the underlying mechanisms through which growth mindset and grit positively impact education.

## Introduction

Positive education advocates for both the well-being and academic performance of students (Seligman et al., 2009). In practice, it fosters positive character traits and cognitions of students to help them achieve not only a higher level of well-being but also better academic performance (Park, 2004). Various positive education programs have been developed to foster these character strengths and cognitions (Waters, 2011; Norrish et al., 2013; Adler, 2016). There is empirical evidence that these programs improve students’ academic performance (Seligman et al., 2009; Adler, 2016). In particular, research and intervention programs on growth mindset and grit are fast growing (Duckworth, 2016). Yet the mechanisms through which growth mindset and grit affect academic performance remain largely unclear. This study aims to fill in these gaps.

### Growth Mindset

Growth mindset, a concept initially developed as a person’s implicit theory of intelligence (Dweck and Leggett, 1988), develops from the belief that an individual’s intelligence is largely malleable (Dweck, 2000). In contrast, fixed mindset is founded upon the theoretical assumption that an individual’s intelligence is mostly immutable. In subsequent research, Dweck (2006) expanded this concept beyond intelligence to apply to personal qualities and abilities, including character strengths and skills. Generally speaking, people who possess a growth mindset tend to see ability as something that can be incrementally developed over time while those who possess a fixed mindset tend to see ability as a fixed, unchangeable entity (Yeager and Dweck, 2012).

Zhang et al. (2017) reviewed the research on the role of students’ mindsets in their academic performance. Most studies found that mindset plays a causal role in academic performance. For example, Mueller and Dweck (1998) found that praising fifth graders for intelligence, which was intended to instill the fixed mindset, performed worse than praising them for effort, which was intended to instill the growth mindset. Various intervention programs have been implemented to improve students’ academic performance through fostering their growth mindset. Blackwell et al. (2007) found that teaching 7th graders about growth mindset can protect them from a further decline in their grades. In another study conducted by Aronson, Fried, and Good, African American college students who were encouraged to see intelligence as malleable rather than fixed achieved better grades than those in the control group (Aronson et al., 2002).

### Learning Motivations

Learning motivations are “the motives…that regulate learners’ study behavior” (Vansteenkiste et al., 2006, p. 19). Self-Determination Theory (SDT; Ryan and Deci, 2000b) differentiates types of learning motivations by the degree to which these motivations are autonomous or controlled. According to SDT, intrinsic motivation is a type of motivation high in autonomy in that people are engaged in an activity for the sake of the activity itself. For example, the fun of learning new things, the interest and curiosity to explore the unknown, and the optimal experience of flow in the activity (Csikszentmihalyi, 1997) are all examples of intrinsic motivations for engaging in an activity.

In contrast, extrinsic motivation is derived from goals that are external to the activity itself. SDT further differentiates extrinsic motivation into four types based on the degree to which this motivation has been internalized (Ryan and Deci, 2000a). The least autonomous type of extrinsic motivation is *external* regulation, which is driven by external rewards or punishments, like monetary rewards for academic achievements and physical punishment for bad exam scores. Another type of extrinsic motivation, *introjected* regulation, is best described as a partially internalized motivation in that it is regulated by a personal desire to affirm one’s ego while still being driven by the external pressure of obtaining the approval of others. Examples of introjected regulation include learning motivated by trying to avoid the disappointment of one’s parents or studying fueled by the belief that one’s self-esteem is contingent upon one’s exam performance.

The other two external motivations, *identified* regulation and *integrated* regulation, are more internalized and integrated into one’s self, and hence more autonomous. People exhibiting an identified regulation style of extrinsic motivation engage in an activity because they accept the value of that activity as personally important as dictated by the goals they endorse. For example, the belief that learning is important. People driven by an integrated regulation style of extrinsic motivation, on the other hand, go further in integrating the activity into other aspects of one’s self such that, for example, they are motivated to learn because of their self-identity as a good learner.

External and introjected regulation styles of extrinsic motivation are classified as controlled motivation styles, whereas identified regulation, integrated regulation, and intrinsic motivation are considered autonomous motivation styles (Ryan and Deci, 2000a). There is ample and solid empirical evidence demonstrating that autonomous learning motivation is positively associated with students’ well-being and academic performance. Conversely, controlled learning motivation is associated with depressive symptoms, mental problems, school disaffection, and academic setbacks (Chia et al., 2016).

### Grit

Grit is defined as “perseverance and passion for long-term goals” (Duckworth et al., 2007, p. 1087), and it’s a character quality that can consistently predict success (Duckworth, 2016). Since it consists of both “perseverance of effort and consistency of interests over time” (Von Culin et al., 2014, p. 1), grit is highly correlated with but goes beyond Conscientiousness, one of the Big Five Personality Factors (Duckworth et al., 2007). Empirical research has consistently shown a positive association between grit and learning outcomes. For example, grittier junior students in high school were more likely to graduate from high school even after controlling for their academic conscientiousness, school motivation, and standardized test scores (Eskreis-Winkler et al., 2014). For Black male college students in a predominantly White institution, grit explained 24% of the variance in their grades (Strayhorn, 2014). Grit scores of undergraduate university students were also positively correlated with GPAs, and this relationship became even stronger when SAT scores were held constant (Duckworth et al., 2007).

### Mediating Roles of Learning Motivations Between Growth Mindset and Grit

There are rich and dynamic relationships between growth mindset, learning motivations, and grit. First, growth mindset fosters autonomous motivations, and fixed mindset fosters controlled motivations. Growth mindset makes people view attributes as malleable through effort and facilitates a higher sense of control (Dweck and Leggett, 1988). According to SDT, the degree to which people perceive the significance of personal choices can impact the degree of autonomy of their motivations (Deci and Ryan, 1985). Therefore, people with growth mindset tend to have more autonomous motivations that enable them to improve their attributes through effort. On the contrary, people with fixed mindset view attributes as fixed and uncontrollable. They have a more controlled form of attribution style that would lead to more controlled motivations.

Second, the type of motivations can influence grit through pathways of both perseverance and passion. Ryan and Connell (1989) measured the learning motivations of elementary school students from urban, suburb as well as rural areas, and found that their external regulation were mostly negatively associated or uncorrelated with effort and enjoyment of learning, while introjected, identified regulation and intrinsic motivation were mostly positively associated with effort and enjoyment of learning. Von Culin et al. (2014) examined the motivational correlates of grit for long-term goals and found that grit was positively associated with *engagement* and *meaning*, and negatively associated with *pleasure*. Since engagement overlaps with intrinsic motivation, meaning regulates people through self-identification and self-integration, and pleasure is typically an external goal, the more autonomous one’s motivation is, the grittier this person might be.

Based on the prior research, we propose a mediation model in which learning motivations mediate the relationship between growth mindset and grit. Yet there might exist other mediators between growth mindset and grit. For example, Duckworth (2016) speculated that people with growth mindset tended to have a more optimistic explanatory style (Peterson and Steen, 2002), which would lead to higher grit. Therefore, we further hypothesize that the paths in our mediating model are mostly partial.

### Influence of Age and Gender

Prior research has demonstrated positive correlations between the above mentioned variables and students’ academic performance regardless of age or gender. In a review of research on participants ranging in age from 4 years old to university student age, Dweck (2006) found that growth mindset was positively associated with better academic performance in all age groups. Autonomous learning motivation has also been found to be associated with better learning behaviors in elementary and middle school students. Furthermore, controlled learning motivation was found to be associated with less optimal learning behaviors in the same students (Ryan and Deci, 2017). Grit has been found to predict the success of students in not only elementary and middle school but also in spelling bee competitions and even in the U.S. Military Academy (Duckworth, 2016).

Similarly, the literature mentioned above also demonstrates that the positive impact of growth mindset, intrinsic learning motivation, and grit on academic performance is consistently felt by both boys and girls. Futhermore, some research has found that the development of positive character traits and coginitions could help close the academic gender gap perpetuated by the stereotype that boys are better math learners than girls. In a field experiment testing this hypothesis, students in a group that were introduced to growth mindset saw a disappearance of the gender differential in math performance on a follow-up exam: both girls and boys did better than they did on the previous exam, and the improvement in girls’ scores was significantlty greater than that of the boys (Good et al., 2003). Zeng et al. (2016) tested the mediating role of resilience between growth mindset and school engagement among 1,279 Chinese primary schools and middle schools. They divided students into three age groups: under 12, between 13 and 15, and 16 and over. They found that the hypothesized mediation model in all three age groups, but the direct effects of growth mindset on school engagement in the 13-to-15 group were less significant than the other two age groups.

Therefore, we hypothesize that relationships between growth mindset, learning motivations, and grit are largely consistent across gender and age groups.

### The Current Study

In light of past findings summarized above, the current study tests a mediating model in which learning motivations partially mediate the association between growth mindset and grit across different gender and age groups. This model has been implied by prior research but never empirically tested. Due to the nature of the model, we used Structural Equation Modeling (SEM) to conduct our analysis.

## Materials and Methods

### Participants and Procedure

Participants were recruited from one public primary school and one public middle school in the city of Tianjin, China. A total of 1,842 students (884 males and 958 females) from third to ninth grade participated in this study. The average age of these students was 11.74 years old, representing a range from 8 to 17 years of age. Informed consent was obtained from participants.

### Measures

The measures we used in this study were translated into Chinese by two graduate students majored in psychology. Translated measures were then back-translated by two other graduate students. Another graduate student majored in psychology checked to ensure the Chinese versions of the measures matched in meaning with the corresponding English versions.

### Growth Mindset

The Growth Mindset Inventory (Dweck, 2006) was used to measure participants’ tendency to have thoughts in line with a growth mindset (vs. a fixed mindset). The inventory consists of eight items. An example item is the statement, “You can learn new things, but you can’t really change your basic level of talent.” Each item, which had been translated into Chinese before being administered to participants, was accompanied by a 5-point response Likert scale. A confirmatory factor analysis with these items produced an acceptable fit: χ^2^/df = 4.02, *RMSEA* = 0.07, *GFI* = 0.90, *TLI* = 0.90, *CFI* = 0.92. The Cronbach’s alpha for the scale was 0.75.

### Learning Motivations

We administered the Academic version of the Self-Regulation Questionnaire (SRA-Academic, Ryan and Connell, 1989) to measure each participant’s learning motivations. The questionnaire describes four learning-related activities, such as doing homework, and provides eight possible answer choices for why a participant would engage in that particular activity. Among the answer choices are responses that correspond to external regulation, introjected regulation, identified regulation, and intrinsic learning motivation. For example, “Because I’ll get in trouble if I don’t” (External), “Because I want the teacher to think I’m a good student” (Introjected), “Because I want to understand the subject” (Identified), “Because I enjoy doing my homework” (Intrinsic). A response corresponding to an integrated regulation was not included in the questionnaire due to very strong similarities between responses corresponding to integrated regulation and responses corresponding to identified regulation. Participants were asked to rate their agreement with each answer choice according to a 5-point Likert scale. It’s important to note that the four types of learning motivations are measured independently in this questionnaire as SDT posits that learning motivations are often intertwined, that is co-existing, rather than mutually exclusive. In this study, a confirmatory factor analysis produced an acceptable fit: χ^2^/df = 3.15, *RMSEA* = 0.05, *GFI* = 0.95, *TLI* = 0.93, *CFI* = 0.96. The Cronbach’s alphas for external regulation, introjected regulation, identified regulation, and intrinsic motivation were 0.85, 0.81, 0.86, and 0.89 respectively.

### Grit

The Short Form Grit Scale (Duckworth and Quinn, 2009) was used in this study to measure participants’ levels of grit. The scale included eight items. For example, one item consisted of the statement, “I start whatever I begin”. Participants were asked to respond by indicating on a 5-point Likert scale their level of agreement with each statement. A confirmatory factor analysis with these items produced an acceptable fit: χ^2^/df = 4.49, *RMSEA* = 0.07, *GFI* = 0.90, *TLI* = 0.90, *CFI* = 0.90. The Cronbach’s alpha of this scale was 0.73.

### Procedure

Research for this study was approved by the Human Research Ethics Committee of Tsinghua University. We also obtained the consent of the school administration, teachers, and students of both the public primary school and the public middle school from which we recruited participants. The participants were notified that all of their responses would only be accessible to the research group. The questionnaires were administered via an online survey. The students answered the survey on school-owned computers in the respective school’s computer room in the 2nd week of September 2017.

### Data Analysis

All the data were entered and sorted in SPSS. First, a confirmatory factor analysis was conducted to test common method bias. Next, analyses of the descriptive statistics and correlations contained within the data were calculated with SPSS. Third, SEM was adopted to analyze mediation effects using the Amos. Lastly, SPSS macro PROCESS with bootstrapping techniques was used to further test and calculate the mediating effects of variables. The effect was significant at the 95% CI. In our statistical analysis, age and gender were included as control variables in order to investigate their potential influence on mediating effects among variables.

### The Control and Test of Common Method Bias

Since all data was collected through questionnaires, common method bias was necessary to test for. Though some techniques (e.g., assuring the respondent of protection of his anonymity) have been adopted to control for this bias, we conducted a confirmatory factor analysis to further ensure the reliability of our research results. Analysis testing the hypothesis that a single factor can account for all of the variance in the data (Podsakoff et al., 2003) revealed a poor model fit (χ^2^/df = 20.43, RMSEA = 0.52, *TLI* = 0.55, *CFI* = 0.62), which indicates that there were no serious biasing effects on estimates of the relationships between constructs.

## Results

### Correlation Analyses

Pearson correlation coefficients were first calculated to examine the relationships among the investigated study variables (see **Table 1**). Gender had no or very small correlations with all variables. Age was negatively correlated with growth mindset and grit, with effect sizes between small and medium. It was not correlated with any of the learning motivations. There existed significant correlations between growth mindset, learning motivations, and grit, except those between growth mindset and introjected regulation and between grit and introjected regulation.

**Table 1:** Correlation coefficients of the variables.

	1	2	3	4	5	6	7	8
(1) Gender	–							
(2) Age	0.03	–						
(3) Growth Mindset	−0.02	−0.15^∗∗∗^	–					
(4) External regulation	0.06^∗^	−0.05^∗^	−0.33^∗∗∗^	–				
(5) Introjected regulation	0.05^∗^	−0.03	−0.03	0.68^∗∗∗^	–			
(6) Identified regulation	0.02	−0.01	0.35^∗∗∗^	0.25^∗∗∗^	0.49^∗∗∗^	–		
(7) Intrinsic Motivation	−0.01	−0.05^∗^	0.38^∗∗∗^	0.09^∗∗^	0.38^∗∗∗^	0.75^∗∗∗^	–	
(8) Grit	−0.09^∗∗^	−0.22^∗∗∗^	0.51^∗∗∗^	−0.27^∗∗∗^	0.04	0.46^∗∗∗^	0.49^∗∗∗^	–

*^∗^p <0.05; ^∗∗^p < 0.01; ^∗∗∗^p < 0.001.*

### Mediating Model Analyses

**FIGURE 1:**
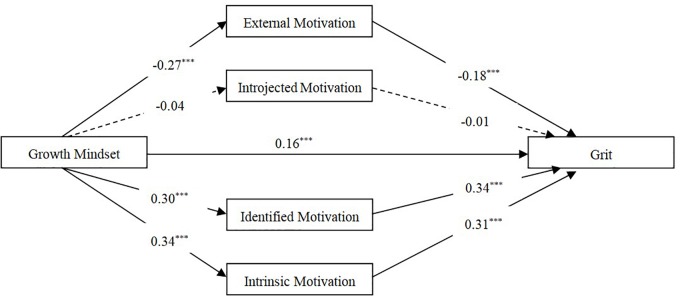
Results of SEM (standardized estimates for paths). ^∗∗∗^*p* < 0.001.

Structural equation modeling was used to test the results of our correlation analyses according to our proposed mediating model, which posits that growth mindset influences grit through the mediating effects of learning motivations (external, introjected, identified, and intrinsic, respectively). Our model results revealed that external regulation, identified regulation, and intrinsic motivation could play significant mediating roles in the relationship between growth mindset and grit (see **Figure 1**). In fact, three significant mediating paths were found connecting growth mindset to grit: 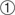 growth mindset – external regulation – grit, 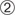 growth mindset – identified regulation – grit, 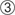 growth mindset – intrinsic motivation – grit.

Then, Hayes’ (2013) SPSS macro PROCESS, a bootstrap program, was used to further test and calculate the mediating effects found in our structural equation model. It was found that all the three mediating paths did not include 0 in the 95% confidence interval, which means that each mediating path was significant. The total mediating effect value was 0.27; these seven mediating paths accounted for 62.94% of the total effect of growth mindset on grit, which is the ratio of indirect effects to the total effects (the predictive value of independent variable on dependent variable). The effect values for each mediating path are presented in **Table 2**. Gender and age were both included as control variables in these analyses.

**Table 2:** Bootstraping analysis of the mediating effects.

Indirect effect	Value	Bootstrap SE	BootstrapLLCI	BootstrapULCI	Relative value
Indirect effect 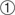	0.05	0.02	0.01	0.11	13.05%
Indirect effect 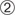	0.10	0.01	0.04	0.15	23.96%
Indirect effect 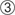	0.11	0.01	0.05	0.17	24.09%

*^∗^p < 0.05*

## Discussion

This study attempts to investigate how the variables of growth mindset, learning motivations, and grit are specifically related to each other. The results support our hypotheses that specific types of learning motivation partially mediate the relationship between growth mindset and grit. The structural equation model we applied to our data demonstrated that having a growth mindset predicts higher degree of autonomy in students’ learning motivations, which in turn positively impacts students’ grit.

To the best of our knowledge, the paths in our mediating model had not been investigated before. Hochanadel and Finamore (2015) have speculated about the causal relationship between growth mindset and grit, and Duckworth (2016) has advocated for instilling growth mindset in children in order to foster their grit. No empirical research in the field, however, has examined the underlying mechanisms that connect growth mindset to grit. A central argument of SDT is that mindsets that foster an individual’s sense of control can facilitate more autonomous types of motivation (Deci and Ryan, 1985). Subsequently, the more autonomous one’s motivations are, the more likely one is to make an effort to persevere and maintain interest in the current activity in which one is engaged (Ryan and Connell, 1989; Ryan and Deci, 2000a). Our finding that learning motivations mediate the relationship between growth mindset and grit provides empirical evidence of the abovementioned theoretical relationships.

One notable outlier among the various learning motivations in our mediating model was introjected regulation, which didn’t play any mediating role. In fact, it was not correlated with growth mindset or grit either. However, since the introjected regulation is somewhat internalized – not as integrated as the identified regulation but more autonomous than the external regulation – it is in line with the theoretical predictions of SDT. This duality of introjected regulation has also been demonstrated in empirical research; introjected regulation was positively associated with effort of learning in a correlational pattern similar to that of autonomous motivation styles, but it was also similar to external motivation style in that it was positively associated with anxiety (Ryan and Connell, 1989).

This study also examined the potential influence of age and gender on these relationships. The results showed that gender was not correlated with any of the investigated variables. Age was negatively correlated with growth mindset, and grit, but not correlated with any specific learning motivations. Our additional finding that the paths in our mediating model were significant even after controlling for gender and age demonstrates that the relationship between growth mindset and grit mediated by learning motivations holds true regardless of gender or age for upper primary and middle school students.

In summary, the findings of this study support the use of a mediating model for explaining the relationships among growth mindset, learning motivations, and grit. This model, which hitherto had never been directly tested, has important implications in both research and practice. It is one of the first empirical studies to link learning motivations as understood in terms of autonomy in Self-Determination Theory to commonly investigated variables in positive education like growth mindset and grit. Evidence for the existence of such an explanatory model calls for future research on whether mechanisms of positive education can be better understood if assessed through SDT. For example, Dweck (2000) believed the impact of mindset was mediated by perceived competence, which is defined by SDT as the basic psychological need that facilitates both the well-being and autonomous learning motivation of students (Chen et al., 2014). Future research, therefore, including on the role of perceived competence in the relationship between growth mindset and learning motivations, should be conducted in order to determine the validity of an SDT-based explanatory framework in positive education.

This study has demonstrated that students who possess a growth mindset tend to find more self-directed and autonomous forms of motivations to learn, thereby increasing their overall grit. These findings highlight the critical importance of teaching growth mindset to primary and middle school students. Once students hold the belief that their intelligence, ability, and other attributes can be improved through their own efforts, they become less prone to the external manipulation of others, and gain a better sense of self through motivating themselves by values, meaning, self-identity, and passion. They will be more likely to persevere in the face of challenges and less likely to give up pursuing an interest in the face of a variety of temptations. Furthermore, the benefits of fostering such a mindset are as far reaching as increased academic performance over the long term. Positive education intervention programs that can effectively foster growth mindset, therefore, need to be designed and implemented in primary and middle schools to increase the well-being and academic performance of students.

Furthermore, this study highlights the importance of learning motivations in education. It replicates the findings of prior research that not all types of learning motivations are good in the long term. The external regulation of extrinsic learning motivation likely leads to less. Educators need to apply strategies to encourage autonomous learning motivations of students and refrain from using external conditions to regulate students. These strategies include autonomy-supportive teaching, needs-supportive teaching, and directing students’ attention to autonomous goals and learning processes (Ryan and Deci, 2017).

### Limitations

The current study faces several limitations. Firstly, this study didn’t consider cultural factors. Though Self-Determination Theory has been supported by many empirical cross-cultural studies (Chirkov et al., 2010), the relationships between some of the studied variables here may be moderated by the different types of self-construal associated with individuals of Eastern (vs. Western) cultural traditions (Markus and Kitayama, 1991). For example, since the self-construal of Chinese students tends to be more interdependent, they may consider expectations of others as expectations of their own, which converts introjected regulation into identified regulation. Future research is needed to examine the relationships we studied here in cross-cultural contexts.

Secondly, since this study is cross-sectional, the mediating model is insufficient for determining any causal relationships that may exist among growth mindset, learning motivations, and grit. More research utilizing experimental, prospective, and longitudinal approaches are needed to identify specific causal (as opposed to just correlational) relationships among the study variables.

Lastly, since grit only moderately correlates with academic performance, our findings are limited in their direct implications for positive education, a discipline ultimately focused on improving the well-being and academic performance of students. Future research, therefore, can supplement our findings by investigating the predictive power of growth mindset on actual academic achievement directly while still taking into account the possible roles learning motivations and grit may play.

## Conclusion

This study found that learning motivations partially mediate the relationship between growth mindset and grit. It study provides insight into the underlying mechanisms behind the positive effects of growth mindset and grit on positive education from the perspective of Self-Determination Theory. As for practical implications, it calls for the design of positive education interventions targeted at fostering students’ growth mindset.

## Author Contributions

YZ and GN contributed equally to this paper. YZ, GN, and GZ designed the study. GN and HH collected and analyzed the data. YZ, GN, and LX wrote the manuscript. KP and FY supervised the study and edited the final draft of the manuscript.

## Conflict of Interest Statement

The authors declare that the research was conducted in the absence of any commercial or financial relationships that could be construed as a potential conflict of interest.
